# Population Structure and Evolution of Non-O1/Non-O139 *Vibrio cholerae* by Multilocus Sequence Typing

**DOI:** 10.1371/journal.pone.0065342

**Published:** 2013-06-11

**Authors:** Sophie Octavia, Anna Salim, Jacob Kurniawan, Connie Lam, Queenie Leung, Sunjukta Ahsan, Peter R. Reeves, G. Balakrish Nair, Ruiting Lan

**Affiliations:** 1 School of Biotechnology and Biomolecular Sciences, University of New South Wales, Sydney, New South Wales, Australia; 2 School of Molecular Bioscience, University of Sydney, Sydney, New South Wales, Australia; 3 Translational Health Science and Technology Institute, Gurgaon, Haryana, India; Beijing Institute of Microbiology and Epidemiology, China

## Abstract

Pathogenic non-O1/non-O139 *Vibrio cholerae* strains can cause sporadic outbreaks of cholera worldwide. In this study, multilocus sequence typing (MLST) of seven housekeeping genes was applied to 55 non-O1/non-O139 isolates from clinical and environmental sources. Data from five published O1 isolates and 17 genomes were also included, giving a total of 77 isolates available for analysis. There were 66 sequence types (STs), with the majority being unique, and only three clonal complexes. The *V. cholerae* strains can be divided into four subpopulations with evidence of recombination among the subpopulations. Subpopulations I and III contained predominantly clinical strains. PCR screening for virulence factors including *Vibrio* pathogenicity island (VPI), cholera toxin prophage (CTXΦ), type III secretion system (T3SS), and enterotoxin genes (*rtxA* and *sto*/*stn*) showed that combinations of these factors were present in the clinical isolates with 85.7% having *rtxA*, 51.4% T3SS, 31.4% VPI, 31.4% *sto*/*stn* (NAG-ST) and 11.4% CTXΦ. These factors were also present in environmental isolates but at a lower frequency. Five strains previously mis-identified as *V. cholerae* serogroups O114 to O117 were also analysed and formed a separate population with *V. mimicus*. The MLST scheme developed in this study provides a framework to identify sporadic cholera isolates by genetic identity.

## Introduction

*Vibrio cholerae* is best known for its capacity to cause pandemic cholera and continues to be a major health threat, highlighted by a recent cholera outbreak in Haiti [1], [2]. With the exception of the O139 Bengal strain, which is known to be a derivative of the 7^th^ pandemic causing epidemic cholera, all major outbreaks and pandemic cholera have been of the O1 serogoup [3], [4]. However, many other O serogroups have been reported for cases of *V. cholerae* associated with diarrhoea in various parts of the world and have been linked with sporadic outbreaks of cholera-like diseases [5]–[10]. Some strains are distributed globally, for example toxigenic O141 strains have been isolated from diverse geographical regions including the USA, Spain, Taiwan and India [8]. Non-O1/non-O139 sporadic outbreaks have been quite common in Bangladesh and India [7], [11]–[13] and have also occurred in Peru [5], Thailand [14] and more recently the USA [9], [10].

Symptoms of infection due to pathogenic non-O1/non-O139 *V. cholerae* strains range from mild gastroenteritis to violent diarrhoea which resembles cholera elicited by the pandemic O1 *V. cholerae* strains [15]. However, in most cases, patients suffer a less severe form of the disease than those infected by O1 *V. cholerae* strains [16]–[18].

The presence of cholera toxin (CTX) and toxin co-regulated pilus (TCP) is characteristic of pandemic isolates and both were initially found only in O1 toxigenic clones. However, many non-O1/non-O139 strains have been found to carry one or both virulence factors [12], [19]–[21]. Some strains such as the O141 strains carry both *Vibrio* Pathogenicity Island (VPI) and cholera toxin phage (CTXΦ) [8]. Other strains carry only VPI or CTXΦ, or VPI plus a partial CTX prophage [22]–[26]. However, none of the isolates studied by Sharma *et al.* [7] has the VPI or CTXΦ. Only two isolates, one O8 clinical and one O11 environmental, of the 34 isolates from 17 serogroups examined by Chakraborty *et al.* [13] contained *tcpA* and *ctx* genes. Other virulence-associated genes such as the *rtxA* and the *sto/stn* genes, which encode the repeat-like toxin (RtxA) - a cytotoxin [27] and the heat-stable enterotoxin (NAG-ST), respectively [28], [29], have also been observed in some of these strains [8], [30]–[32]. More recent studies showed that a type III secretion system (T3SS) is present in some non-O1/non-O139 isolates and appears to be an important virulence factor for these isolates. The T3SS translocates a number of T3SS effectors to the host cell such as VopF and VopE which interfere with host cell signalling pathways [33], [34]. A functional T3SS has been shown to be essential for the pathogenicity of the non-O1/non-O139 strain AM-19226 [35]. Environmental isolates have also been reported to carry one or more of the virulence factors [30], [31], [36], [37].

Strain description up to now primarily uses serogrouping based on O antigen, and serogroup has been used as a major indicator in assessing virulence potential. There are over 200 serogroups [38] and at least 19 have been reported to be associated with sporadic cholera including O10, O12, O26, O31, O37, O53 and O75 [12], [13], [15], [36], [39]–[41]. Some isolates from these non-O1/non-O139 sporadic cholera cases have been characterised by DNA fingerprinting [7], [13] and gene sequencing [39]. However, the genetic background of the clinical and environmental isolates is diverse and a sequence-based study of relationships of the isolates is much needed. In this study, multilocus sequence typing (MLST) was applied to 35 clinical and 20 environmental non-O1/non-O139 *V. cholerae* isolates, including 15 isolates from sporadic cases in Bangladesh from 1998 to 2001, to examine their relationships to each other and with the O1 toxigenic and pandemic strains. There were no seven-gene MLST schemes available when this study was commenced, although one has been published recently [42].

## Materials and Methods

### Bacterial Isolates Used

The 55 *V. cholerae* isolates used were either clinical isolates or environmental isolates (Table 1). Fifteen of the 55 isolates were sporadic isolates from Bangledesh from 1998 to 2001 while others were from various countries with the majority being O antigen reference strains and were from the collection by T. Shimada, National Institute of Health, Japan.

**Table 1:** *Vibrio cholerae* and other *Vibrio* spp. isolates analysed in this study.

Strain Name	Original Strain Name	Category	Serogroup	Location (year)	VPI†	T3SS§	CTX	*rtxA*	*NAG-ST*	*adk*	*gyrB*	*mdh*	*metE*	*pntA*	*purM*	*pyrC*	ST	Clonal Complex (CC)#	Subpopulation¶
M2565	AQ 24151	Clinical	O94	Bangladesh (2001)	−	+	−	+	−	26	5	2	1	31	19	45	50	CC2	I
M2549	AO 823	Clinical	O12	Bangladesh (1999)	−	−	−	+	−	12	23	2	49	30	1	40	9	Singleton	I
M553	141-94	Environmental	O70	Germany (1994)	+	−	−	+	−	17	5	14	50	31	14	23	22	Singleton	I
M1121	208	Clinical	O27	Thailand (1992)	+	−	−	+	−	19	9	12	23	9	1	5	26	Singleton	I
M1576		Clinical	O124	India (1981)	−	−	−	+	+	2	7	8	22	39	1	45	31	Singleton	I
M1052	334-72	Clinical	O26	Phillipines (1972)	−	−	−	+	−	24	5	14	50	31	14	45	39	Singleton	I
M2559	AQ 9921	Clinical	O2	Bangladesh (2001)	−	+	−	+	−	26	23	2	50	31	1	45	43	Singleton	I
M2547	AN 26575	Clinical	O44	Bangladesh (1998)	−	−	−	+	−	26	3	1	50	14	18	45	44	Singleton	I
M2561	AQ 13192	Clinical	O145	Bangladesh (2001)	−	+	−	+	−	26	5	1	8	14	14	45	45	Singleton	I
M2560	AQ 11489	Clinical	O15	Bangladesh (2001)	−	+	−	+	−	26	5	14	50	31	14	2	46	Singleton	I
M535	#82	Environmental	O1	Thailand (1992)	−	+	−	+	+	26	5	14	50	31	15	45	47	Singleton	I
M2564	AQ 18953	Clinical	O24	Bangladesh (2001)	−	−	−	+	−	27	5	9	21	30	6	45	51	Singleton	I
M2556	AP 20403	Clinical	ND	Bangladesh (2000)	−	+	−	+	−	26	5	2	1	31	17	45	49	CC2	II
M536	#35	Environmental	O1	Thailand (1992)	−	+	−	+	+	1	15	19	15	24	5	14	3	Singleton	II
M1562	AU256	Environmental	O110	Japan (1992)	−	+	−	+	+	1	41	21	4	2	16	43	4	Singleton	II
M1095	27973	Clinical	O77	India (1976)	+	−	−	+	+	1	42	4	60	20	1	12	5	Singleton	II
M2563	AQ 16457	Clinical	O24	Bangladesh (2001)	−	−	−	+	−	12	23	2	6	30	17	40	10	Singleton	II
M1105	984-81	Clinical	O89	India (1981)	−	+	−	+	+	12	5	3	33	31	18	26	11	Singleton	II
M555	905-93	Environmental	O97	Argentina (1993)	+	−	−	+	+	13	40	17	46	3	9	39	12	Singleton	II
M559	179-94	Environmental	O52	Denmark (1994)	−	−	−	−	−	14	1	14	44	6	9	39	13	Singleton	II
M1071	1154-74	Clinical	O49	India (1974)	−	−	−	+	−	14	14	4	53	22	17	35	14	Singleton	II
M1098	1421-77	Clinical	O80	India (1977)	+	+	−	+	+	14	23	6	47	11	1	36	15	Singleton	II
12129	Genome*	Environmental	O1	Australia (1985)	+	+	−	+	−	14	31	20	39	16	1	13	16	Singleton	II
M1118	577-88	Clinical	O105	China (1988)	+	+	+	−	−	14	38	14	43	8	21	11	17	Singleton	II
M560	190-94	Environmental	O39	Denmark (1994)	−	+	−	+	−	14	6	14	7	3	20	47	18	Singleton	II
M548	ATCC33653	Environmental	O25	Germany (1993)	+	+	−	+	+	14	6	29	11	25	1	24	19	Singleton	II
VL426	Genome	Environmental	non-O1/non-O139	UK	−	−	−	+	−	15	29	16	18	10	1	22	20	Singleton	II
M1049	169-68	Clinical	O22	Phillipines (1968)	−	−	−	+	−	18	5	14	40	17	1	1	24	Singleton	II
M1051	14438-62	Clinical	O24	Phillipines (1962)	+	−	−	+	−	19	23	14	12	1	1	6	25	Singleton	II
RC385	Genome	Environmental	O135	Chesapeake bay (1998)	−	−	−	+	+	2	10	18	45	4	8	48	27	Singleton	II
MZO-2	Genome	Clinical	O14	Bangladesh (2001)	−	−	−	+	−	2	23	15	42	18	13	10	28	Singleton	II
AM-19226	Genome	Clinical	O39	Bangladesh (2001)	+	+	−	+	−	2	5	13	19	6	6	5	29	Singleton	II
1587	Genome	Clinical	O12	Peru (1994)	+	−	−	+	−	2	6	29	2	25	1	3	30	Singleton	II
M556	921-93	Environmental	O74	Argentina (1993)	−	−	−	+	−	22	39	10	38	3	17	15	36	Singleton	II
M1078	1463-76	Clinical	O57	India (1976)	−	−	−	+	+	24	5	1	6	39	18	2	38	Singleton	II
M1036	8394-62	Clinical	O7	India (1962)	−	+	−	−	+	24	5	14	55	30	1	44	40	Singleton	II
M1575	345-81	Clinical	O123	India (1981	−	−	−	−	+	24	5	14	55	7	1	45	41	Singleton	II
M1593	243-93	Clinical	O141	India (1993)	+	+	+	+	+	25	5	22	16	21	8	11	42	Singleton	II
V51	Genome	Clinical	O141	US	+	+	+	+	−	25	5	22	16	21	8	11	42	Singleton	II
TM11079-80	Genome	Environmental	O1	Brazil (1980)	+	−	−	+	−	26	5	14	54	28	14	45	48	Singleton	II
M549	1085-93	Environmental	O37	Germany (1993)	−	−	−	+	−	4	23	28	13	25	1	9	65	Singleton	II
MZO-3	Genome	Clinical	O37	Bangladesh (2001)	−	−	−	+	−	4	23	28	13	25	1	9	65	Singleton	II
M563	370-94	Environmental	O81	Korea (1994)	−	+	−	−	+	5	26	14	34	2	4	47	66	Singleton	II
M2552	AO 21097	Clinical	O27	Bangladesh (1999)	−	+	−	+	−	6	4	14	35	15	3	46	67	Singleton	II
M1618	N1 (V523)	Environmental	non-O1/non-O139	Australia (1977)	+	−	+	+	+	7	11	4	17	23	1	35	68	Singleton	II
M2129	ATCC 25872	Clinical	O37	Czechoslovakia (1965)	+	−	+	+	+	7	11	4	17	23	1	35	68	Singleton	II
M2130	S-21	Clinical	O37	Sudan (1968)	+	−	+	+	+	7	11	4	17	23	1	35	68	Singleton	II
V52	Genome	Clinical	O37	Sudan	+	−	+	+	−	7	11	4	17	23	1	35	68	Singleton	II
M1086	981-75	Clinical	O65	India (1975)	+	−	−	+	−	7	12	14	37	12	1	36	74	Singleton	II
CT5369-93	Genome	Environmental	non-O1/non-O139		−	−	−	+	−	7	24	30	59	3	1	45	76	Singleton	II
M562	369-94	Environmental	O10	Korea (1994)	−	−	−	+	−	8	30	27	36	3	9	39	78	Singleton	II
TMA21	Genome	Environmental	non-O1/non-O139	Brazil (1993)	+	+	−	+	−	9	25	3	41	6	1	5	79	Singleton	II
N16961	Genome	7th pandemic	O1	Bangladesh (197X)	+	−	+	+	−	7	11	4	37	12	1	20	69	CC1	III
M2140	SIMP/77	Australian toxigenic	O1	Australia (1977)	+	−	+	+	+	7	11	4	37	12	1	37	70	CC1	III
M2141	M4287/77	Australian toxigenic	O1	Australia (1977)	+	−	+	+	+	7	11	4	37	12	1	37	70	CC1	III
BX330286	Genome	Australian toxigenic	O1	Australia (1986)	+	−	+	+	+	7	11	4	37	12	1	37	70	CC1	III
M802	M66-2/Makassar 759 (Genome)	pre-7th pandemic	O1	Indonesia (1937)	+	−	+	+	−	7	11	4	37	12	1	38	71	CC1	III
M1616	O395 (Genome)	6th pandemic	O1	India (1965)	+	−	+	−	+	7	11	4	9	12	1	38	73	CC1	III
M796	4808	US Gulf Coast	O1	USA (1978)	+	−	+	+	+	7	2	4	37	12	1	38	75	CC1	III
M2562	AQ 14875	Clinical	O49	Bangladesh (2001)	−	+	−	+	−	1	1	4	48	29	1	41	1	CC3	III
M2553	AP 2007	Clinical	O49	Bangladesh (2000)	−	+	−	+	−	1	1	4	48	29	17	41	2	CC3	III
M2554	AP 9172	Clinical	O49	Bangladesh (2000)	−	+	−	+	−	1	1	4	48	29	17	41	2	CC3	III
M2555	AP 14558	Clinical	O49	Bangladesh (2000)	−	+	−	+	−	1	1	4	48	29	17	41	2	CC3	III
M2558	AQ 5961	Clinical	O49	Bangladesh (2001)	+	+	−	+	−	1	1	4	48	29	17	41	2	CC3	III
M1619	N19 (V58)	Environmental	non-O1/non-O139	Australia (1977)	+	−	+	+	+	1	8	15	5	13	1	7	6	Singleton	III
M1035	B4202-64	Clinical	O5	Phillipines (1964)	−	+	−	−	+	11	27	11	56	19	1	4	8	Singleton	III
M1092	113-79	Clinical	O73	India (1979)	−	+	−	+	−	11	27	11	56	19	1	4	8	Singleton	III
623-39	Genome	Environmental	non-O1/non-O139	Bangladesh (2002)	+	−	−	+	−	20	41	5	3	18	2	4	32	Singleton	III
M550	1089-93	Environmental	O99	Germany (1993)	−	+	−	+	−	3	28	11	56	19	12	4	54	Singleton	III
M561	366-94	Environmental	O74	Korea (1994)	−	−	−	+	−	7	36	7	10	38	1	42	77	Singleton	III
M554		Environmental	O83	Germany (1994)	+	−	−	+	+	10	13	25	57	34	10	25	7	Singleton	IV
M1565	AU105	Environmental	O113	Japan (1995)	−	+	−	+	−	16	33	26	31	34	11	26	21	Singleton	IV
M1563	AU291	Environmental	O111	Japan (1993)	−	+	−	−	+	17	5	25	32	36	11	29	23	Singleton	IV
M1093	429-79	Clinical	O75	India (1979)	+	−	−	−	−	21	37	23	58	35	7	28	33	Singleton	IV
M557	928-93	Environmental	O6	Argentina (1993)	−	−	−	−	−	22	32	24	20	33	9	27	34	Singleton	IV
M558	929-93	Environmental	O66	Argentina (1993)	−	−	−	−	−	22	32	24	20	5	1	27	35	Singleton	IV
M1560	AU124	Environmental	O108	Japan (1990)	+	−	−	−	−	23	34	26	57	37	10	25	37	Singleton	IV
M2548	AN 28767	Clinical	O37	Bangladesh (1998)	−	−	−	−	−	26	3	1	N/A‡	14	18	N/A			
M2550	AO 13987	Clinical	O8	Bangladesh (1999)	N/A	N/A	N/A	N/A	N/A	N/A	1	11	N/A	N/A	N/A	N/A			
M2551	AO 21097	Clinical	O27	Bangladesh (1999)	−	−	−	−	−	34	41	33	58	26	31	N/A			
M547	ATCC33653	*V. mimicus*								31	18	41	52	40	23	33	56		
M1566	246-79	*V. mimicus*	O114	USA (1979)						32	20	39	29	44	30	19	58		
M1567	523-80	*V. mimicus*	O115	USA (1980)						32	21	38	26	46	25	34	59		
M1115	559-88	*V. mimicus*	O101	China (1988)						32	22	42	27	41	27	18	60		
M1569	381-82	*V. mimicus*	O117	Japan (1982)						33	19	35	30	42	29	31	61		
M1568	980-78	*V. mimicus*	O116	USA (1978)						34	17	34	28	43	31	16	62		
RC341	Genome	*V. metecus*								28	35	31	14	27	22	8	52		
M552			O103	Germany (1993)						37	42	43	47	27	34	50	95		
RC586	Genome	*V. parilis*								29	35	33	51	26	32	21	53		
VM223	Genome	*V. mimicus*		Brazil						30	16	36	62	40	24	30	55		
MB451	Genome	*V. mimicus*		Bangladesh						31	22	40	24	41	28	17	57		
SX-4	Genome	*V. mimicus*		China (2009)						32	21	38	26	46	25	34	59		
VM573	Genome	*V. mimicus*		USA (1990s)						32	21	38	26	46	25	34	59		
VM603	Genome	*V. mimicus*		Brazil (1990s)						35	19	37	25	45	26	32	63		
CMCP6	Genome	*V. vulnificus*								36	43	32	61	32	33	49	64		

* Genome strains 1587 (GenBank Accession No. AAUR00000000); 623-39 (Accession No. AAWG00000000); AM-19226 (Accession No. AATY00000000); BX330286 (Accession No. ACIA00000000); CMCP6 (Accession No. AE016795.3); CT5369-93 (Accession No. ADAL00000000); M66-2 (Accession No. CP001233); MB451 (Accession No. ADAF00000000); MZO-2 (Accession No. AAWF00000000); MZO-3 (Accession No. AAUU00000000); N16961 (Accession No. AE003852); M1616/O395 (Accession No. CP000626); RC341 (Accession No. ACZT00000000); RC385 (Accession No. AAKH00000000); RC586 (Accession No. ADBD00000000); SX-4 (Accession No. ADOO00000000); TM11079-80 (Accession No. ACHW00000000); TMA21 (Accession No. ACHY00000000); V51 (Accession No. AAKI00000000); V52 (Accession No. AAKJ00000000); VL426 (Accession No. ACHV00000000); VM223 (Accession No. ADAJ00000000); VM573 (Accession No. ACYV00000000); VM603 (Accession No. ACYU00000000); 12129 (Accession No. ACFQ00000000).

† Two primer pairs were used as previously described in Tay *et al*. [44] to amplify divergent *tcpA* genes.

§ Two primer pairs were used for typing the type III secretion system (T3SS), one for *vcsC2* and one for *vcsV2*.

‡ N/A: Data not available.

# Clonal complex (CC) as defined by eBURST analysis.

¶ Subpopulations are derived from STRUCTURE analysis.

### MLST Genes and Primers

Seven genes were selected from the 26 housekeeping genes that we used previously to determine the evolutionary relationships of the pandemic clones [43]. Three criteria were used to select seven genes: 1) both chromosomes are represented with five (*gyrB, mdh, adk, metE, purM*) and two (*pntA* and *pyrC*) genes from chromosomes one and two, respectively; 2) the genes are evenly distributed around the chromosomes; 3) These genes are present in related *Vibrio* species. In order to design primers that will be able to identify all of *V. cholerae* strains, primers were based on conserved regions among the sequences of more closely related *Vibrio* spp. The primers for the seven housekeeping genes (*gyrB*, *mdh*, *adk*, *metE*, *purM*, *pntA* and *pyrC*) are shown in Table 2.

**Table 2:** Primers used in this study.

Gene	Gene Product	Direction*	Oligonucleotide Sequence (5' à 3')	Reference
*adk*	adenylate kinase	F	CATCATTCTTCTCGGTGCTC	This study
		R	AGTGCCGTCAAACTTCAGGTA	
*gyrB*	DNA gyrase subunit B	F	GTACGTTTCTGGCCTAGTGC	This study
		R	GGGTCTTTTTCCTGACAATC	
*metE*	methionine synthase	F	CGGGTGACTTTGCTTGGT	This study
		R	CAGATCGACTGGGCTGTG	
*mdh*	malate dehydrogenase	F	ATGAAAGTCGCTGTTATTGG	This study
		R	GCCGCTTGGCCCATAGAAAG	
		R	TAGCTTGATAGGTTGGG	This study
*pntA*	pyridine nucleotide transhydrogenase	F	CTTTGATGGAAAAACTCTCA	
		R	GATATTGCCGTCTTTTTCTT	This study
		F	GGCCAGCCCAAAATCCT	
*purM*	phosphoribosyl-formylglycinamide cyclo-ligase	F	GGTGTCGATATTGATGCAGG	This study
		R	GGAATGTTTTCCCAGAAGCC	
*pyrC*	Dihydroorotase	F	ATCATGCCTAACACGGTTCC	This study
		R	TTCAAACACTTCGGCATA	
*ctxAB*	cholera toxin	F	CTCAGACGGGATTTGTTAGGCACG	[69]
		R	TCTATCTCTGTAGCCCCTATTACG	
*rtxA*	repeat like toxin	F	GCGATTCTCAAAGAGATGC	[27]
		R	CACTCATTCCGATAACCAC	
*sto*/*stn* (NAG-ST)	heat-stable toxin	F	CCTATTCATTAGCATAATG	[12]
		R	CCAAAGCAAGCTGGATTGC	
*tcp*	toxin co-regulated pili	F1	GTGACTGAAAGTCATCTCTTC	[44]
		R1	AATCCGACACCTTGTTGGTA	
		F2	ATATGCAATTATTAAAACAGC	
		R2	TTATTATTACCCGTTGTCGG	
*vcsC2*	inner membrane protein	F	GGAAAGATCTATGCGTCGACGTTACCGATGCTATGGGT	[12]
		R	CATATGGAATTCCCGGGATCCATGCTCTAGAAGTCGGTTGTTTCGGTAA	
*vcsV2*	ATPase	F	ATGCAGATCTTTTGGCTCACTTGATGGG	[12]
		R	ATGCGTCGACGCCACATCATTGCTTGCT	

### PCR Assay and DNA Sequencing

Each PCR reaction included 2.5 µl of DNA template (approx. 20 ng), 0.5 µl (30 pmol/µl) of each forward and reverse primer (Table 2), 0.5 µl 10 mM dNTPs, 5 µl 10x PCR buffer (500 mM KCl, 100 mM Tris-HCl pH 9.0, 1% Triton® X-100 and 15 mM MgCl_2_), 0.25 µl (1.25 U) Taq polymerase (Promega) and MilliQ water to a total volume of 50 µl. PCR cycles were performed in a Hybaid PCR Sprint Thermocycler (Thermo Analysis Biocompany, Hybaid Limited, UK) with the following conditions: initial DNA denaturation for 2 min at 94°C; followed by DNA denaturation for 15 sec at 94°C, primer annealing for 30 sec at 50°C and polymerization for 1 min 30 sec at 72°C for 35 cycles, with a final extension of 5 min at 72°C. PCR products were verified on EtBr stained agarose gels before purification using sodium acetate/ethanol precipitation. The PCR sequencing reactions contained BigDye™ and were done as recommended by the manufacturer (Applied Biosystems). We sequenced both the 5′ and 3′ ends of the amplicons. Unincorporated dye terminators were removed by ethanol precipitation. The reaction products were separated and detected by gel electrophoresis using Automated DNA Sequence Analyzer ABI3730 (Applied Biosystems) at the Ramaciotti Centre (University of New South Wales, Sydney, Australia).

### PCR Detection of Virulence Genes

We tested for the presence of the *tcpA* gene using two sets of primers as previously used to amplify divergent *tcpA* genes [44] and two primer pairs for the T3SS, one for *vcsC2* and one for *vcsV2* [12]. For both VPI and T3SS, if one primer pair was positive, we interpreted it as a positive result, respectively. We used only one primer pair for the other genes (*ctxAB, rtxA* and *sto/stn*) (Table 2).

### Bioinformatic Analysis

The PHRED-PHRAP-CONSED [45] program package was utilised for sequence editing. ClustalW [46] and MULTICOMP [47] were used for multiple sequence alignment and comparison. PHYLIP [48] was used to generate phylogenetic trees and bootstrap values. SplitsTree version 3.2 [49] was used to create a network structure using the Neighbour-net algorithm and uncorrected “p“ distance [50]. The ratio of recombination (ρ) to mutation (θ) in different loci was estimated using a composite likelihood program LDhat version 2.2 [51].

Sequence variants of the seven genes were designated as alleles and the combination of seven alleles constitutes an allelic profile. Isolates with identical allelic profiles were assigned to the same sequence type (ST). The STs were analysed using eBURST [52] to determine the presence of clonal complexes (CCs). Clonal complexes are groups of closely related STs, which shares six loci to at least one other ST within the group, and are descendants of a recent common ancestor. The founder of a CC is defined as the ST that differs from the largest number of other STs at only a single locus.

STRUCTURE version 2.2 [53], which implements a Bayesian approach for analysis of MLST data, was used to assess the possibility that the isolates were derived from a finite number of prior “cryptic” populations with varying degrees of admixture. The number of populations, *K*, was determined under the “admixture” model and in each simulation run, the Markov Chain Monte Carlo (MCMC) simulation of 30,000 iterations gave the posterior probability of *K* following a burn-in of 10,000 iterations. Different values of *K* were run multiple times and the *K* value that generated the highest posterior probability was used as the probable number of ancestral populations. The assignment of an isolate to a particular population was done under the linkage model.

The GenBank accession numbers are KC894962-KC895395. Sequences are deposited into the PubMLST database using the Bacterial Isolate Genome Sequence platform [54] and are accessible at http://pubmlst.org/vcholerae/.

## Results and Discussion

### MLST Scheme for *V. cholerae*

We developed a new MLST scheme for *V. cholerae* that was based on fragments of seven housekeeping genes. The gene fragment sequences ranged in length from 416 bp –591 bp, for a total of 3,217 bp for concatenated sequences. We applied this MLST scheme to 55 non-O1/non-O139 *V. cholerae* strains representing 43 O antigen types and one strain of unknown O type (not tested). Most O antigens were represented by a single isolate but six types were represented by two to six isolates. As detailed below, an additional five *V. mimicus* strains, which were initially identified as *V. cholerae,* were included. Two other presumptive *V. cholerae* isolates, M552 and M2551, were found to be of other *Vibrio* species based on MLST.

Twenty-seven different O antigens were found in the 35 clinical isolates. Fifteen were isolated in Bangladesh between 1998 and 2001, including nine O antigens (one isolate was not typed), of which two were O24 isolates from the same year and five were O49 isolates over a 2 year period. The 20 environmental isolates represented 18 different O antigens.

### Sequence Variation

PCR and sequencing of the seven housekeeping genes were initially done for 63 presumptive *V. cholerae* isolates. Sequences were obtained for all seven genes for 60 isolates. For strain M2548, two genes (*metE* and *pyrC*) failed and for strain M2551, one gene (*pyrC*) failed. For M2550, four genes (*pyrC*, *adk*, *pntA* and *metE*) failed to amplify a product and the strain was excluded from further analysis. Sequence data from 17 genome sequenced strains (excluding pandemic and related genome strains that were already represented) were also included for comparison. The most conserved gene was *purM* with maximum and average pairwise percentage differences of 2.94 and 0.78%, respectively while the most variable gene was *pyrC* with the highest maximum and average pairwise percentage differences of 16.48% and 6.50%, respectively.

### Allelic Profiles, Sequence Types and Clonal Complexes

There were 66 STs, six of which were represented by multiple isolates, while the remaining STs were found in a single isolate. The STs with most isolates were ST2 and ST68, both of which contained four isolates. We used the definition of six out of seven shared alleles for a CC and identified only three CCs. CC1 was the largest containing seven STs and also included the two pandemic clones. The remaining two CCs were CC2 (ST49 and ST50) and CC3 (ST1 and ST2), all containing clinical isolates. The founder of CC1 was ST71 which was represented by M66-2, a pre-seventh pandemic strain, while CC2 and CC3 contained only two member STs and the founders could not be determined.

Four of the 17 genome strains had the same ST as a strain sequenced in this study. V51 was identical to M1593. Both were of O141 serogroup but were isolated from different locations in different years and were members of a known widespread toxigenic clone [8]. Genome strain V52 was identical to three other strains, M1618, M2129 and M2130. These strains were all O37 clinical isolates. Both V52 and M2130 were isolated in Sudan while M2129 was from Czechoslovakia. Interestingly, M1618 was an environmental isolate from Australia. The genome strain MZO-3 was identical to M549, both of which were of O37 serogroup. We previously sequenced 26 genes including the seven MLST genes from M549 [43]. The additional 19 genes were also identical to those of MZO-3. Lastly, genome strain BX330286, an O1 isolate, was found to be of the same ST as two other isolates, M2140 and M2141. All three isolates were from Australia and are toxigenic.

M2551 was the most divergent and clearly belonged to a different species. M552 was most closely related to RC341, the sole member of a new *Vibrio* species, *V. metecus*, proposed by Haley *et al*. [55]. The percentage DNA difference for the seven genes is 3.72% between M552 and RC341 which was far lower than that (8.1%) between M552 and the closest *V. cholerae* strain MZO-2.

### Phylogenetic Relationships and Population Structure of *V. cholerae*

**Figure 1:**
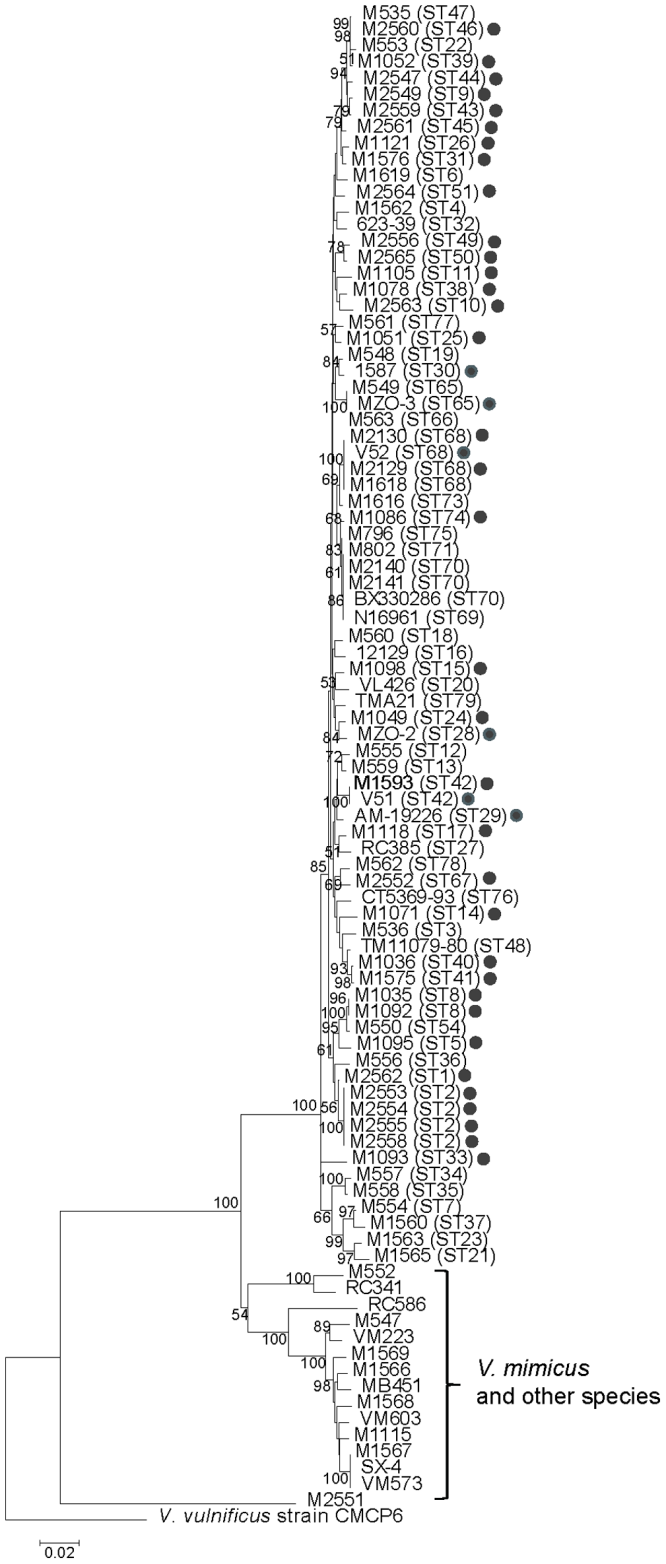
Phylogenetic relationships of *Vibrio cholerae* isolates based on neighbour-joining tree. ST is indicated in bracket after strain name for *V. cholerae*. Non-O1/non-O139 *V. cholerae* strains from clinical sources are marked with a dot. *Vibrio vulnificus* strain CMCP6 was used as an outgroup. Bootstrap values, if greater than 50%, are presented at nodes of the neighbour joining trees.

**Figure 2:**
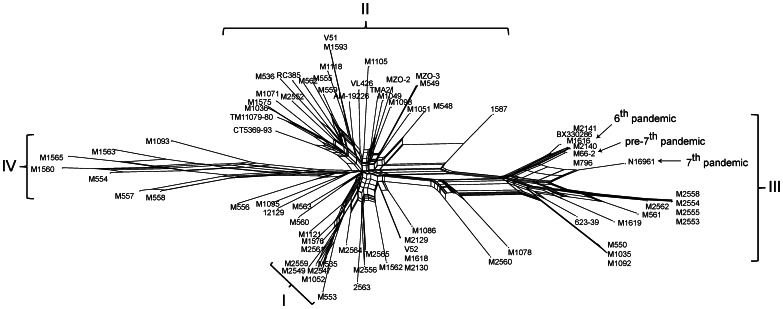
Neighbour-net network of *Vibrio cholerae* isolates analysed in this study. Four subpopulations (I to IV) as determined by STRUCTURE analysis are indicated with curly brackets. Isolates associated with 6^th^, pre-7^th^ and 7^th^ pandemics are indicated with an arrow.

We performed a number of analyses to determine relationships and groupings of the isolates. A neighbour joining (NJ) tree was constructed using *V. vulnificus* strain CMCP6 as an outgroup to show the overall relationships (Figure 1). The NJ tree gave good bootstrap values only for branches with species-level differences. There was low bootstrap support for most branches, which may be due to recombination as *V. cholerae* is known to have a high level of recombination [43], [56]. A bifurcating tree may not be the best representation for their relationships, so a SplitsTree was constructed and showed an extensive network structure (Figure 2), consistent with high levels of recombination.

We further assessed the frequency of recombination using the composite likelihood of recombination rate and mutation rate using LDhat [51]. The estimated rates of recombination (ρ) and mutation (θ) and their ratios for each gene are shown in Table 3. The ρ/θ ratio ranged from 0.465 (in *metE*) to 5.871 (in *adk*) and averaged 2.427 over the seven genes, showing significant levels of recombination. The average rate was much higher than these for *E. coli* [57] and *Salmonella* [58] with an average of 1.531 and 1.258 respectively. We also used the counting method of Feil *et al*. [59] to determine the nature of the changes observed in the three clonal complexes (CC1-CC3), in which the single allele difference between STs within a clonal complex was attributed to either mutation if the difference was a single base or recombination otherwise. There were five recombinational events and one mutational event in total, giving a ratio of 5 to 1. However, since there were only three CCs and only a few member STs within a CC, this estimate was likely to be unreliable. In the study of Salim *et al*. [43], 26 housekeeping genes were sequenced from the pandemic and closely related toxigenic strains, which provided another dataset to estimate the ratio of recombination to mutation. Together with strains added in the current study (see the section on the relationship of M1086 with pandemic lineages below), the 26 housekeeping gene data revealed 14 recombinational events and six mutational events within the two pandemic lineages (excluding the seven recombinational events between the two lineages, see the section on the relationship of M1086 with pandemic lineages below). This gave a recombination to mutation ratio of 2.333 to 1, which was similar to the estimate by the composite likelihood method above. This ratio can be compared with the estimates using the same method of other organisms including *Y. pseudotuberculosis* of a ratio of 1∶1 [60], *Neisseria meningitidis* of 10∶1 and *Staphylococcus aureus* of 1∶15 [61]. The results suggest that *V. cholerae* has a weakly clonal population structure.

**Table 3:** Estimates of mutation (θ) and recombination (ρ) rates.

	ρ	θ	ρ/θ
*adk*	0.089	0.015	5.871
*gyrB*	0.060	0.023	2.578
*mdh*	0.017	0.019	0.882
*metE*	0.017	0.036	0.465
*pntA*	0.053	0.020	2.622
*purM*	0.061	0.015	3.956
*pyrC*	0.031	0.050	0.619
Mean	0.047	0.026	2.427

**Figure 3:**
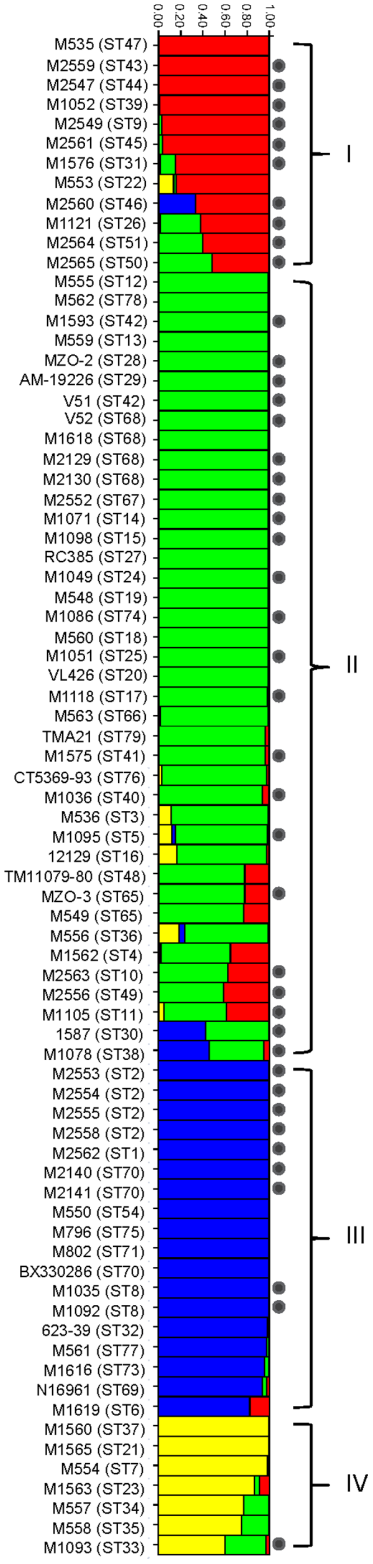
STRUCTURE analysis of *Vibrio cholerae* isolates including genome strains. The four subpopulations are colour-coded with red, green, blue and yellow for subpopulation I, II, III and IV respectively. Each isolate has been allocated to a subpopulation. Isolates were identified by strain name with ST in brackets on the left. Mosaic colours for an isolate indicate mixed population origin from respective populations of matching colour. Y-axis represents percentage of population assignment. Non-O1/non-O139 *V. cholerae* strains from clinical sources are marked with a dot.

We then used the Bayesian statistics tool, STRUCTURE to determine the population structure of the 66 *V. cholerae* STs (77 isolates). The most striking finding is that the *V. cholerae* isolates fell into four subpopulations that were designated as subpopulations I, II, III and IV (Figure 3). These were most clearly seen in the STRUCTURE plot (Figure 3), which we used to allocate isolates to the four subpopulations. The four subpopulations were also well demarcated in the SplitsTree (Figure 2).

Subpopulation I contained 12 STs (12 isolates). Subpopulations II and III contained 34 STs (39 isolates) and 13 STs (19 isolates), respectively. Subpopulation IV contained seven STs (7 isolates). Subpopulations I and III were predominantly clinical isolates. Subpopulation III contained the pandemic clones and closely related isolates. Five isolates from sporadic cases from Bangladesh also belonged to this subpopulation which will be discussed below. Subpopulation II contained slightly more clinical isolates than environmental isolates. All except one isolate in subpopulation IV were of environmental origin although the subpopulation was small with only seven isolates. The majority of the genome sequenced strains fell into subpopulation II. Thus, more genomes should be sequenced from the other subpopulations to assess overall genomic diversity of the species.

The ancestry of each isolate/ST was estimated as the sum of probable sources from each of the presumed ancestral subpopulations over all polymorphic nucleotides. Thirty-one STs had no imports from other populations while 36 isolates (35 STs) contained alleles from other ancestral subpopulations (Figure 3); with 23 and 13 isolates having imports from one and two other ancestral subpopulations, respectively. The proportion of nucleotides from one or more minority subpopulations varied from 1% to 51%. Therefore, there were considerable gene flows between subpopulations.

It is interesting to note that the subpopulation assignment had two anomalies. ST68 (M1618, M2129, M2130 and V52) and ST74 (M1086) were closely related to pandemic strains (see below) but were assigned to subpopulation II rather than subpopulation III contrary to our expectations. This may be due to the different number of informative bases in different genes which influenced the subpopulation assignment.

### Comparison with Environmental Isolates Typed by an Alternative MLST Scheme

Since the completion of this study, an MLST scheme has been applied to a large set of environmental isolates [42]. The two schemes share only two genes, which means that a direct seven-gene sequence based comparison was not feasible. However, we can compare the populations based on strains common to the two studies. There were 12 non-O1/non-O139 genome sequenced strains from Chun *et al*. [62] which we added to the environmental dataset. A STRUCTURE analysis divided this set of isolates into five subpopulations (data not shown). The genome sequenced strains all fell into one subpopulation which was the largest subpopulation. We also compared the isolates using the two genes (*mdh* and *gyrB*) common to the two studies (Figure S1). There were no clear demarcations of the subpopulations that were designated for the isolates analysed in this study. All except subpopulation IV were distributed across the NJ tree. The distribution of isolates in the tree showed that the two studies complemented each other in extending the genetic diversity.

### Detection of Virulence Associated Genes in Sporadic Cholera Isolates

Previous studies have shown that sporadic cholera isolates may carry VPI or both VPI and CTX factors [12], [19]–[21]. Rtx toxin, T3SS and NAG-ST have also been reported to contribute to their virulence [27]–[29]. We used PCR to screen isolates for the presence of genes encoding these virulence factors. Excluding the genome strains, there were 35 clinical isolates. The most common virulence factor carried was RtxA at 85.7%, followed by T3SS at 51.4%, VPI and NAG-ST, each at 31.4% and CTX at 11.4%. All isolates carried at least one of these five virulence factors and one strain (M1593, O141) was positive for all five factors. Three isolates were positive for both VPI and CTX and an additional seven isolates were only positive for VPI. Clearly, the pathogenic mechanisms for non-O1/non-O139 strains were variable and diverse. For the environmental isolates, the most common virulence factor carried was also RtxA at 70%, followed by NAG-ST at 40%, T3SS and VPI each at 35% and CTX at 10%. Therefore, the number of virulence factors carried by environmental isolates was generally lower. This was also reflected in 15% of the environmental isolates not carrying any of the five virulence factors. This observation suggests that some strains isolated from the environment may not have a diarrhoeagenic potential in humans, while others do have the potential to cause diarrhoea.

Previous studies have reported that many strains carry VPI and/or CTX, including O27 [26], O37 [63], O44 [26], O64 [64], O65 [24] and O141 [8], [65] strains. Some other strains carry only VPI or CTXΦ or VPI plus a partial CTX prophage [22]–[24]. This study showed a similar picture for distribution of VPI and CTX, with 31.4% of the clinical isolates carrying VPI, although all our CTX positive isolates were also positive for VPI.

The virulence factors appeared to be quite mobile. Within the same ST, there were differences in the presence of the virulence factors. One of the four ST2 isolates was VPI positive. Our ST42 strain M2556 was negative for both VPI and CTX while the genome sequenced strain, V51, of the same ST carried both VPI and CTX. Of the two ST8 isolates, one carried only RtxA while the other carried only NAG-ST.

### Further Resolution of Relationships of M1086 and Pandemic and Closely Related Isolates based on 26 Housekeeping Genes

M1086 (ST74) was closest to V52 (ST68) together with three other ST68 strains (M1618, M2129 and M2130). Note that of the four ST68 isolates, V52 and M2130 (original name S-21) were isolated in the same year (1968) and from the same location (Sudan) and were probably from the same outbreak. M2129 was an O37 strain isolated in 1965 from Czechoslovakia while M1618 (unknown serogroup) was isolated from Australia in 1977 from an environmental source.

The NJ tree from the MLST data presented an anomaly regarding the relationship of the pandemic and closely related strains. V52 and M1086 were closely related but were placed on different branches, well separated on the seven gene MLST tree from the pandemic strains. However, a previous analysis based on 26 housekeeping genes showed that the two pandemic clones grouped together [43] and this grouping has been confirmed by genome sequence data [56], [62]. The genome study by Chun *et al*. [62] defined two clades with the 7^th^ pandemic lineage as phylocore genotype (PG) clade 1 and the 6^th^ pandemic lineage as PG clade 2 [62]. The genome tree placed the O37 strain V52 in PG clade 2.

**Figure 4:**
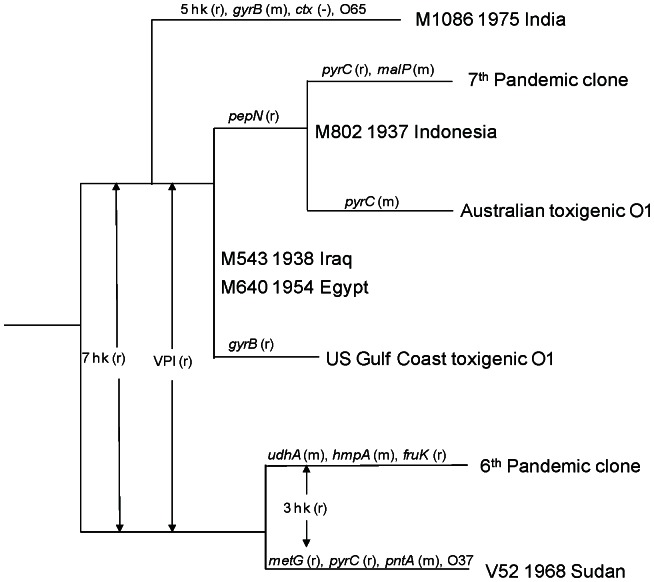
Relationships of M1086 and pandemic related strains based on minimum spanning tree (MST). MST was constructed using allelic difference of 26 housekeeping (hk) genes which are either resulted from recombination (r) or mutation (m). Events were marked on the branches with gene symbol (r or m) or for multiple genes as number of hk genes affected. M1086 and V52 were analysed in this study. See Salim *et al.* [43] for details of others. Changes of *Vibrio* pathogenicity island (VPI) and cholera toxin (CTX) are also indicated. Sequence data were M1086 from this study, V52 from Chun *et al*. [62] and others from Salim *et al.* [43]. Strain names, year of isolation and place of isolation were shown except for the pandemic strains and Australian and US Gulf toxigenic isolates.

We sequenced 19 M1086 genes to complete the 26 gene set that Salim *et al*. [43] sequenced previously for the pandemic and closely related strains for comparison (see Salim *et al*. for the details of the 19 genes), and used the V52 genome sequence to represent ST66. A minimal spanning tree was constructed based on allelic difference rather than nucleotide sequence difference, which placed M1086 in the 7^th^ pandemic lineage as the earliest diverged strain in that lineage (Figure 4). We denoted difference by one base as mutation and by two or more bases as recombination. M1086 differed from the ancestral alleles of that lineage by recombination in five genes (*gppA, hmpA, metG*, *pepN* and *pyrC*) and by mutation in one gene (*gyrB*), with the remaining 21 genes identical. Interestingly, three of the seven MLST genes showed a difference with one gene (*gyrB*) by mutation and two genes (*mdh* and *pyrC*) by recombination, which distorted the true relationship of the strain to the 7^th^ pandemic lineage when seven genes were used. M1086 carried the O65 O antigen, and can thus be considered as a pre-7^th^ pandemic strain that has the O1 antigen replaced, if we assume the ancestral strain was O1. M1086 was positive for VPI but negative for CTX which may have been lost. M1086 had also lost mannose-sensitive haemagglutinin pilin gene based on PCR detection (data not shown). Both pandemic lineages can now be seen to have at least one occasion of O antigen replacement in their early divergence, in the 6^th^ pandemic lineage by O37 and the 7^th^ pandemic lineage by O65.

### Diversity of Sporadic Cholera Isolates from Bangladesh Region

We included in this study 18 non-O1/non-O139 isolates of 11 different serogroups isolated over four years from Bangladesh, with complete MLST data for 15 isolates from nine different serogroups. MLST data showed that none of these isolates was closely related to pandemic strains or derivatives of the pandemic clones by O antigen switching. MLST showed that the five O49 isolates from two different years were closely related, with four typed as ST2 and one as ST1. The two STs differed in one locus due to a single base change and were grouped together in CC3. Two isolates, typed as ST50 and ST49, belonged to CC2. One isolate belonged to serogroup O94 while the other was not typed for serogroup and may also be an O94 serogroup isolate. Two O24 isolates (M2563 and M2564) had two different STs differing by five loci, suggesting that they were not closely related. The remaining six isolates of different O serogroups had unique STs and differed from each other by three to six loci. These results suggest that there were multiple clones of *V. cholerae* causing sporadic cholera in Bangladesh with some being more prevalent.

### Relationship with *V. mimicus* Strains

Five strains M1568, M1569, M1566, M1115 and M1567 were grouped together with our known *V. mimicus* strain M547 and five genome sequenced *V. mimicus* strains (VM603, VM573, VM223 MB451 and SX-4) [55], [66]–[68] (Figure 1), showing that these five “*V. cholerae*” strains were in fact *V. mimicus* that were initially misidentified. Maximum and average pairwise differences amongst the 11 isolates were 3.42% and 1.94%, respectively, while the maximum and average pairwise differences between *V. cholerae* and *V. mimicus* were 11.49% and 8.21%. Thus, the two species were well separated by sequence variation. Sucrose fermentation is commonly used to distinguish these species and we found them to be sucrose-negative as expected for *V. mimicus*.

Two of the five *V. mimicus* genome strains, VM573 (Clinical, USA, 1990s), VM223 (Environmental, Brazil) and SX-4 (Clinical, China, 2009) were identical to our M1567 (Clinical, USA, 1980) for all seven MLST genes and belong to ST59. All three strains were VPI+ and CTX+, suggesting that this ST is widespread as a cholera agent.

We also performed a STRUCTURE analysis with the 11 *V. mimicus* strains included and as expected, they were separated as a population of their own (data not shown). Interestingly, the *V. mimicus* population did not show any evidence of obtaining genes from the *V. cholerae* subpopulations and *vice versa*, which implies that there is clear species boundary for recombination. Given the high recombination rate in *V. cholerae*, this observation is surprising. However, it may be due to the small number of *V. mimicus* isolates sampled and/or the method of detection. There were small segments in the *pyrC* gene that were evidently of recombinant origin. A 60 bp segment in *V. mimicus* strains M547 and M1568 was likely to have been imported from *V. cholerae* which was not detected by STRUCTURE.

### Conclusions

The identification of non-epidemic *V. cholerae* has primarily been based on O antigen as there were no real alternatives. MLST provided a framework to identify sporadic cholera isolates by their genetic characteristics. Sporadic cholera isolates are diverse. However, some were isolated in multiple locations or years, suggesting that these strains with repeated isolations are important pathogens and may have epidemic or pandemic potential. Much larger scale sampling will be required to monitor non-O1/non-O139 sporadic cholera clones.

We have shown in this study that the *V. cholerae* population can be divided into four subpopulations. Subpopulations I and III consisted of mainly clinical isolates, with pandemic clones belonging to subpopulation III. The ratio of recombination rate to mutation rate is 2.427, suggesting that *V. cholerae* is weakly clonal.

There was considerable variability in the proportion of the non-O1/non-O139 clinical isolates to carry virulence factors, with 85.7%, 51.4%, 31.4%, 31.4% and 11.4% of these isolates carrying RtxA, T3SS, VPI, NAG-ST and CTX, respectively. Only one of these isolates had all five virulence genes. These virulence genes were also present in the environmental isolates but at a lower frequency.

*V. mimicus* was well separated from *V. cholerae* by population structure analysis. One ST including two genome sequenced strains carrying VPI and CTX, is an important lineage of *V. mimicus* as a human pathogen.

## Supporting Information

Figure S1 **Phylogenetic relationships of** ***Vibrio cholerae*** **isolates based on neighbour-joining tree using concatenated sequences of two genes (** ***mdh*** **and** ***gyrB*** **) common between this study and the study by Keymer** ***et al*** **.** [42]. *Vibrio vulnificus* strain CMCP6 was used as an outgroup.
(PDF)
