# Supplementary figures and images for: Population Structure and Evolution of Non-O1/Non-O139 Vibrio cholerae by Multilocus Sequence Typing
Source: PLoS One. 2013 Jun 11;8(6):e65342. doi: 10.1371/journal.pone.0065342 (PMC3679125; doi:10.1371/journal.pone.0065342)

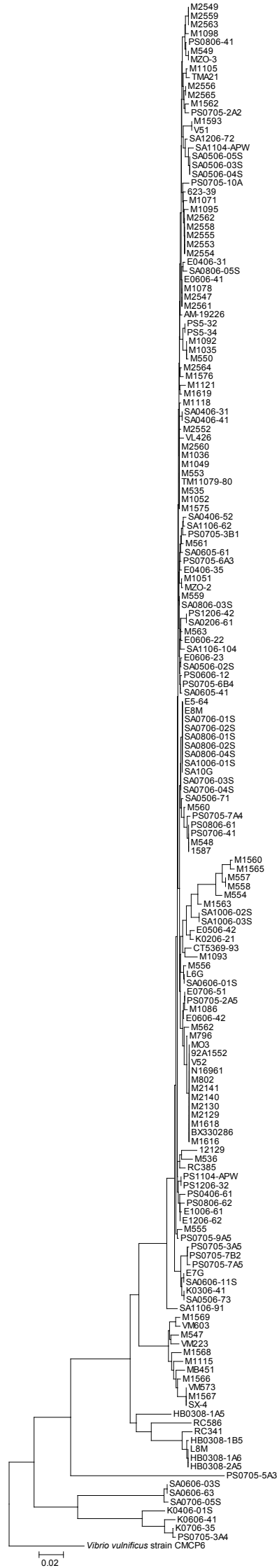

Supplement: Figure S1 — Phylogenetic relationships of Vibrio cholerae isolates based on neighbour-joining tree using concatenated sequences of two genes ( mdh and gyrB ) common between this study and the study by Keymer et al . [42]. Vibrio vulnificus strain CMCP6 was used as an outgroup. (PDF) [file pone.0065342.s001.pdf]
